# Prone Positioning in Non-Intubated Patients With COVID-19 Outside of the Intensive Care Unit: More Evidence Needed

**DOI:** 10.1017/dmp.2020.267

**Published:** 2020-07-27

**Authors:** Alba Ripoll-Gallardo, Luca Grillenzoni, Jordy Bollon, Francesco della Corte, Francesco Barone-Adesi

**Affiliations:** CRIMEDIM – Research Center in Emergency and Disaster Medicine, Università del Piemonte Orientale, Novara, Italy; Nuovo Ospedale Degli Infermi, Biella, Italy; Department of Translational Medicine, Università del Piemonte Orientale, Novara, Italy

**Keywords:** continuous airway pressure ventilation, COVID-19, noninvasive mechanical ventilation, prone positioning

## Abstract

The coronavirus disease (COVID-19) pandemic has brought the Italian National Health System to its knees. The abnormally high influx of patients, together with the limited resources available, has forced clinicians to make unprecedented decisions and provide compassionate treatments for which little or no evidence is yet available. This is the case for the use of noninvasive positive pressure ventilation and continuous airway pressure ventilation, combined with prone position in patients with COVID-19 and acute respiratory distress syndrome treated outside of intensive care units. In our article, we comment on the evidence available, so far, and provide a brief summary of data collected at our health institution in Piedmont, Italy.

Italy is a high-income country; however, the ongoing coronavirus disease (COVID-19) pandemic turned hospitals into low-resource battlefields where the provision of the best level of care for a single patient was no longer feasible and “the best for the most” was the only possible alternative. Even though health facilities have made an unprecedented effort to expand their surge capacity, intensive care units (ICUs) have been under strain with health professionals struggling to provide appropriate care to wave after wave of critically ill patients.

Prone positioning (PP) improves oxygenation and decreases mortality in intubated patients with acute respiratory distress syndrome (ARDS). Even if, theoretically, the same benefits should apply to awake patients, the use of PP in non-intubated patients has seldom been described before the COVID-19 outbreak.^1-4^ Interestingly, as the pandemic continues its spread across the globe, the shortage of ventilators and ICU beds is driving clinicians to increasingly use PP outside of ICUs in an attempt to avoid or delay endotracheal intubation.^5,6^

Sartini et al.^5^ have recently published a small case series of COVID-19-positive patients with mild-to-moderate ARDS treated with noninvasive ventilation (NIV) and PP, reporting that respiratory parameters improved after pronation. Similarly, Elharrar et al.^6^ published a prospective study with 24 patients requiring oxygen supplementation where PP was well tolerated, but had a limited effect on oxygenation. Albeit highly relevant, both investigations presented an important limitation: the follow-up period in both studies was limited to 14 and 10 days, respectively. Therefore, intubation rate and outcomes could not be provided for all patients.

We hereby present data from a retrospective case series of 13 COVID-19-positive patients with moderate-to-severe ARDS treated at Nuovo Ospedale degli Infermi di Biella, a 500-bed suburban hospital in Piedmont, one of the most severely hit Italian regions. Patients received helmet continuous positive airway pressure (CPAP) with 0.6 fraction of inspired oxygen (FiO2) and 10 CMH20 positive end-expiratory pressure (PEEP) and were pronated in general wards if PaO2:FiO2 < 150 mmHg. Endotracheal intubation was performed in case of respiratory failure, hemodynamic instability, or multiorgan failure. PP was maintained as long as it was well tolerated. The Wilcoxon test was used to compare PaO2:FiO2 and the respiratory rate before and after PP. Analyses were performed using Stata 15 Software (Stata Corp, College Station, TX). A *P*-value less than 0.05 was considered statistically significant.

Demographic data, coexisting chronic diseases, respiratory parameters before and after PP, laboratory values, and patient outcomes are reported in Table 1. Mean (SD) age was 66.3 years (7.7 years). Mean (SD) PaO2:FiO2 before PP was 115 (13). Our results showed an improved PaO2:FiO2 compared to baseline in 12 patients (*P* = 0.003). No difference was found in the respiratory rate before and after PP (*P* = 0.20). Only 4 patients (30%) avoided intubation and 6 (46%) survived and were discharged home. Interestingly, the improvement in PaO2:FiO2 appeared to be greater in survivors but didn’t achieve significance (*P* = 0.668).

**TABLE 1:** Demographic, Clinical and Laboratory Data of COVID-19 Patients Treated With CPAP and PP

Patient	1	2	3	4	5	6	7	8	9	10	11	12	13
**Gender**	M	M	M	M	M	M	M	F	M	M	F	M	M
**Age (y)**	69	72	73	68	65	65	59	44	65	69	74	68	70
**BMI**	30	18	27	35	22	33	33	23	29	31	26	38	39
**Coexisting chronic diseases** *	AF, COPD	AH, DM, Asthma	AH	AH, Dyslipemia Hyperuricemia	AH, CHD AF, Anemia DM	AH	AH	N/A	AH, DM, Dyslipemia	Myasthenia gravis	AH	AH	AH, AF
**Time from symptom onset to admission (d)**	7	7	7	3	7	5	7	8	7	7	7	5	10
**Time from admission to CPAP (d)**	1	0	4	0	4	1	3	0	3	5	1	2	1
**Time from CPAP to first PP (d)**	3	2	1	1	0	2	3	1	0	0	1	1	1
**ET**	Yes	Yes	Yes	Yes	Yes	Yes	Yes	No	No	No	Yes	Yes	No
**Time from first PP to ET (d)**	4	1	3	0	0	1	2	N/A	N/A	N/A	9	0	N/A
**Total number of PP before ET**	4	1	6	1	1	2	2	2	1	1	8	1	3
**Time from admission to ET (d)**	8	4	8	1	4	4	8	N/A	N/A	N/A	11	3	N/A
**Respiratory rate before first PP**	38	32	24	28	48	20	38	24	40	40	18	24	34
**Respiratory rate after first PP**	30	21	24	24	48	20	30	32	40	40	18	26	28
**PaO2:FiO2** ** **before first PP**	121	101	108	98	136	121	113	145	118	114	109	109	105
**PaO2:FiO2 after first PP**	126	138	130	101	230	178	230	350	178	162	126	98	116
**Time in PP** *** **(h)**	2	2	3.5	2	0.75	3	3	3	2	1	2.5	3.5	3
**SOFA score**	3	3	3	4	3	3	3	3	4	3	4	4	4
**WBC count /x mmc before first PP** **n.r 4000-10.000**	11 900	12 340	6840	19 830	12 310	13 010	9120	10 570	4090	7440	4934	8080	13 600
**Lymphocyte count x mmc before first PP n.r 1000-3400**	631	629	588	674	566	390	1368	1216	540	841	1030	517	666
**Lymphocyte % n.r 19-48%**	5.3%	5.1%	8.6%	3.4%	4.6%	3%	15%	11.5%	13.2%	11.3%	16.1%	6.4%	4.9%
**D-dimer ug/L before first PP n.r <70**	980	1598	1190	10 966	25 495	3667	3414	1665	980	1268	683	312	336
**Complications of PP**	None	None	None	None	None	None	None	None	None	None	None	None	None
**Outcome**	Discharged	Dead	Dead	Dead	Dead	Dead	Dead	Discharged	Discharged	Discharged	Dead	Discharged	Discharged

* Abbreviations: COVID-19: coronavirus disease 2019; M: male; F: female; BMI: body mass index, calculated as weight in kilograms divided by height in meters squared; COPD: chronic obstructive pulmonary disease; AF: atrial fibrillation; DM: diabetes mellitus; CHD: coronary heart disease; AH: arterial hypertension. NR: normal range; CPAP: continuous positive airway pressure, PP: prone position, ET: endotracheal intubation.

** The normal PaO_2_:FIO_2_ ratio is more than 400 mm Hg; if PaO_2_:FIO_2_ less than 300 mm Hg indicates acute respiratory distress syndrome.

*** For patients with more than one PP cycle, time is expressed as the mean time in PP.

To our knowledge, this is the first study on CPAP with PP in COVID-19 patients. The oxygenation results are consistent with those of Sartini et al.^5^ and other previously published studies^3,4^; however, Sartini et al.^5^ reported that PP improved respiratory rate, while in our case series and that of Scaravilli et al.,^3^ no difference before and after was found. This could be explained by the fact that the mean age of patients in Sartini et al.’s study was 59 years,^5^ whereas in ours and Scaravilli et al.’s^3^ studies, mean ages were 66.3 and 66 years, respectively. Moreover, our patients were more severely ill at the time of the PP, which might have increased the chance of selection bias in Sartini et al.’s^5^ study.

Finally, our results do not suggest a lower intubation or death rate. However, no conclusions can be drawn at the current stage given the retrospective study design, small sample size, lack of control group, and incomplete data of our case series and other published studies.

Importantly, pending results from ongoing randomized controlled trials (NCT04347941, NCT04350723), the outcomes of all COVID-19 patients with ARDS included in small case series and treated with NIV combined with PP would supply more helpful information on whether an improvement in PaO2:FiO2 could translate into avoidance of intubation and increased survival.
